# Dynamic Changes of NCR^−^ Type 3 Innate Lymphoid Cells and Their Role in Mice with Bronchopulmonary Dysplasia

**DOI:** 10.1007/s10753-021-01543-7

**Published:** 2022-02-04

**Authors:** Jiayu Cai, Hongyan Lu, Zhaoliang Su, Lanlan Mi, Suqing Xu, Zhengyang Xue

**Affiliations:** 1 Department of Pediatrics, Affiliated Hospital of Jiangsu University, Zhenjiang, Jiangsu 212000 China; 2 Laboratory of Immunopharmacology, Jiangsu University, Zhenjiang, Jiangsu 212000 China

**Keywords:** bronchopulmonary dysplasia, ILC3, NKP46^−^ILC3, IL-17, IL-22

## Abstract

Inflammation is one of the important pathogenesis of bronchopulmonary dysplasia (BPD). Type 3 innate lymphoid cells (ILC3) play a role in a variety of inflammatory lung diseases. In this study, we established the BPD model by injecting lipopolysaccharide into the amniotic cavity of pregnant mice. Here, we investigated the dynamic changes of ILC3 and NKP46^−^ ILC3 population in lung tissues of mice from BPD and the control groups. Results showed that the proportion of ILC3 and NKP46^−^ILC3 in the BPD group was higher than those of the control group. In addition, the cytokines interleukin-17 (IL-17) and interleukin-22 (IL-22) secreted by ILC3 in this model had also changed that their expression was significantly increased compared with that of the control group. Flow cytometry demonstrated that ILC3 were a rapid source of IL-17. In the anti-CD90 knockdown experiment, we confirmed the alleviation of BPD inflammation in the absence of ILC3. In addition, we injected mice with anti-IL-17 neutralizing antibody, and the results showed that IL-17 could aggravate BPD inflammation. Taken together, ILC3 may play a pro-inflammatory role in BPD by secreting IL-17.

## INTRODUCTION

Bronchopulmonary dysplasia (BPD) is a chronic lung inflammatory disease that usually occurs in premature infants. It is characterized by abnormal alveolar and pulmonary vascular development [1, 2]. With obstetric and neonatal intensive care technology, improvement of auxiliary ventilation strategies, antenatal corticosteroids, and the use of pulmonary surfactant after birth, the survival rate of very low birth weight infants has been upregulated, but morbidity of bronchopulmonary dysplasia has been upregulated simultaneously [3]. Studies have shown that the probability of respiratory diseases in children with BPD is significantly increased [4], which brings a heavy economic burden to society and families. Therefore, it has become an urgent problem to explore the pathogenesis of BPD in depth to guide the early prevention and subsequent treatment.

The etiology of BPD is considered to include lung immaturity, infection and inflammation, mechanical ventilation, oxygen poisoning, etc. In the development of BPD, inflammation is an essential regulatory factor [2], while type 3 innate lymphoid cells (ILC3) have been found to play a proinflammatory role in inflammatory intestinal diseases through secreting cytokines interleukin-17 (IL-17) and interleukin-22 (IL-22) [5]. Besides, the effect of ILC3 on pneumonia has attracted much attention, but their role in BPD remains unclear.

Innate lymphoid cells (ILC) are a class of immune cells with adaptive immune function, which play an important role in tissue repair and homeostasis and mediate immune response in a variety of mucosal tissues [6–8]. They can be classified into three subsets: ILC1, ILC2, and ILC3. All three subsets of ILC are present in the airways of mice and humans. In human lung tissue, about 60% of ILC are ILC3 [9]. ILC3 express retinoic acid–related orphan receptor γt(RORγt) and secrete IL-17, IL-22, granulocyte–macrophage colony-stimulating factor (GM-CSF), and interferon-γ (IFN-γ) in reaction to the stimulation of interleukin-23 (IL-23) or interleukin-1β (IL-1β) [10]. ILC3 play a key role in homeostasis, infection, and inflammation of mucosal barrier. Furthermore, according to the expression of the natural cytotoxicity-triggering receptor (NCR) Nkp46 in mice, ILC3 can be divided into NCR^**−**^ILC3 and NCR^**+**^ILC3 [11, 12]. During the development of immunity, NCR^−^ ILC3 are important for the formation of lymphoid organs, and they mainly cluster with stromal cells, dendritic cells (DCs), B cells in crypts, isolated lymphoid follicles, or mature isolated lymphoid follicles in adult mice [13]. Besides, NCR^−^ILC3 can secrete IL-17, a cytokine that is crucial in fighting fungal infections. However, inappropriate activation of ILC3 has also been shown to induce tissue damage through excessive expression of IL-17, IL-22, and GM-CSF, resulting in accumulation of neutrophils and tissue destruction [14]. For example, in intestinal inflammation, ILC3 can recruit an abundance of monocytes by secreting GM-CSF to induce inflammation and aggravate intestinal inflammation by secreting IL-17 and IFN-γ as well [15], but their role in respiratory diseases remains unclear.

Current studies have shown that ILC3 are involved in a variety of pulmonary diseases, such as bacterial pneumonia, tuberculosis, asthma, chronic obstructive pulmonary disease, and influenza [16–19]. Recent studies have identified that a higher frequency of ILC3 was found in asthma patients compared to healthy controls, as well as elevated IL-17 and IL-22 production secreted by ILC3 [20]. Also, an increase of IL-17 ^+^ ILC3 was found in mice fed a high-fat diet after house dust mite attack [18]. Besides, in studies of cigarette smoke–induced chronic obstructive pulmonary disease in mice, an increase in all ILC populations was found in bronchoalveolar lavage fluid, especially IL-17 ^+^ILC [21], suggesting that ILC3’s rapid secretion of IL-17 and IL-22 is one of the important mechanisms of pulmonary inflammation. However, there are still few studies on the role of ILC3 in BPD IL-17 that can induce the production of inflammatory cytokines such as tumor necrosis factor (TNF), IL-1, and GM-CSF. Besides, it can also recruit neutrophils in inflammation [18]. Thus, we proposed a hypothesis that NCR^**−**^ILC3 may play an important role in BPD by secreting IL-17. Pulmonary inflammation plays a key role in the development of BPD, and chorioamnionitis induced by lipopolysaccharide (LPS) is one of the classical models of BPD in mouse [2]. To identify it, the proportion of ILC3 and the expression of their downstream cytokines in lung tissues were detected in the LPS-induced BPD model.

## MATERIALS AND METHODS

### Animals and Tissue Preparation

C57BL/6 mice at ages of 8 to 12 weeks (weight 19.1 ± 0.6 g) were purchased from the Animal Center of Jiangsu University (Zhenjiang, China). Twenty male and twenty female mice were selected at a ratio of 1:1 into twenty cages. The next day, the vaginal secretions of female mice were smeared, and the mice with sperm-bearing vaginal secretions detected by smear microscopy were recognized as pregnant. The BPD model was replicated by intra-amniotic injection of LPS (200 μg/kg) [2]. Thus, the pregnant mice were randomly divided into the LPS and normal saline group on the 14th day of gestation. Intra-amniotic injection of LPS was administrated in the LPS group, and normal saline was given to the control group. Newborn mice from mothers in the LPS group were set as the BPD group, and those from mothers in the saline group were set as the control group. Five mice in each group were sacrificed on postnatal days 1, 3, 7, and 14, and lung tissues were collected. Mice with similar body weight in the same group came from the same litter. All animal researches were approved by the Animal Center of Jiangsu University.

### Histological Analysis

For lung morphometry, tissues were fixed with 4% paraformaldehyde and embedded in paraffin. After sectioning into 3-µm thick, lung tissues were stained with hematoxylin and eosin and viewed under bright light microscopy using upright fluorescence microscope. Images were captured at × 200 magnification.

### Single Cell Suspension Preparation

Fresh lung tissues of newborn C57BL/6 mice on days 1, 3, 7, and 14 were taken and rinsed with normal saline to remove residual blood. Lung tissues were sectioned into small pieces and ground on a 100-μm cell strainer, with 1 mL phosphate-buffered saline (PBS) added. Then, cells were inhaled into a 1.5-mL EP tube with a 1-mL pipette after filtering through a 70-μm cell strainer. Next, cells were centrifuged at 3500 rpm for 5 min, after which the supernatant was discarded. One milliliter ACK Lysis Buffer was added for erythrolysis on ice for 5 min, and centrifuged at 3500 rpm for 5 min. Finally, the supernatant was disgarded and 100 μL PBS was added to complete the preparation.

### Flow Cytometry

For surface staining, the following mouse monoclonal antibodies were used: FITC-lineage, PE-CD127, PC-5.5-Nkp46, PC5.5-IL-17, and PC-5.5-Nkp46. For intracellular staining, cells were stimulated for 5 h at 37 °C with phorbol-12-myristate-13-acetate (PMA) (50 ng/ml), ionomycin (1 μg/ml), and Brefeldin A (2 μg/ml). The intracellular fixation and permeation buffer were used for fixation and cell rupture, followed by intracellular staining using the following antibody: APC-RORγt. ILC were analyzed using specific markers of ILC populations. ILC3 are defined as Lin-CD127 + RORγt + cell population [11]. At least 1 × 10^5^ cells were collected by CytoFlex flow cytometer.

### Cytokine Measurements

Enzyme-linked immunosorbent assay (ELISA) kits were used to detect the protein levels of IL-17, IL-22, and GM-CSF in lung tissue homogenate. Lung tissues of mice were extracted and rinsed with normal saline to remove the residual blood on postnatal days 1, 3, 7, and 14. Tissue homogenate was prepared by ultrasound. BCA Protein Assay Kit was used to examine the total protein concentration of each tube. Protein content of IL-17, IL-22, and GM-CSF in lung tissues was detected according to the instructions.

### Expansion of ILC3 by Injection of Recombinant IL-23

Studies have shown that IL-23 can stimulate the expansion of ILC3 [22]. Thus, recombinant IL-23 (10 ng) was injected intraperitoneally into newborn mice on postnatal days 7, 9, 11, and 13 to expand ILC3. BPD mice that received normal saline injection were set as the control.

### Blockade of CD90* In Vivo*

In order to knock down the ILC3 in lungs of BPD mice, anti-CD90 antibody (50 μg) was injected intraperitoneally on postnatal days 7, 9, 11, and 13 into newborn mice [23]. BPD mice that received normal saline injection were set as the control. Then, mice were sacrificed on postnatal day 14. ILC3 ratio was detected by flow cytometry and lung tissues were collected for HE staining.

### Blockade of IL-17* In Vivo*

For blockade of IL-17 in BPD mice, anti-IL-17A neutralizing antibody (85 μg) was injected intraperitoneally into newborn mice on postnatal days 7, 9, 11, and 13 [24]. BPD mice that received normal saline injection were set as the control. Then, mice were sacrificed on postnatal day 14, and lung tissues were collected for HE staining.

### Statistics

The data were analyzed by Graphpad Prism 8.0.1 statistical software, expressed as mean ± SEM. One-way ANOVA was used among multiple groups. Multiple comparison between the groups was performed using LSD method. *P* values < 0.05 were considered significant. Significant difference in the data is indicated by asterisk. *P* values < 0.05 are indicated by a single asterisk.

## RESULTS

### Pathological Changes in Lung Tissue

**Fig. 1:**
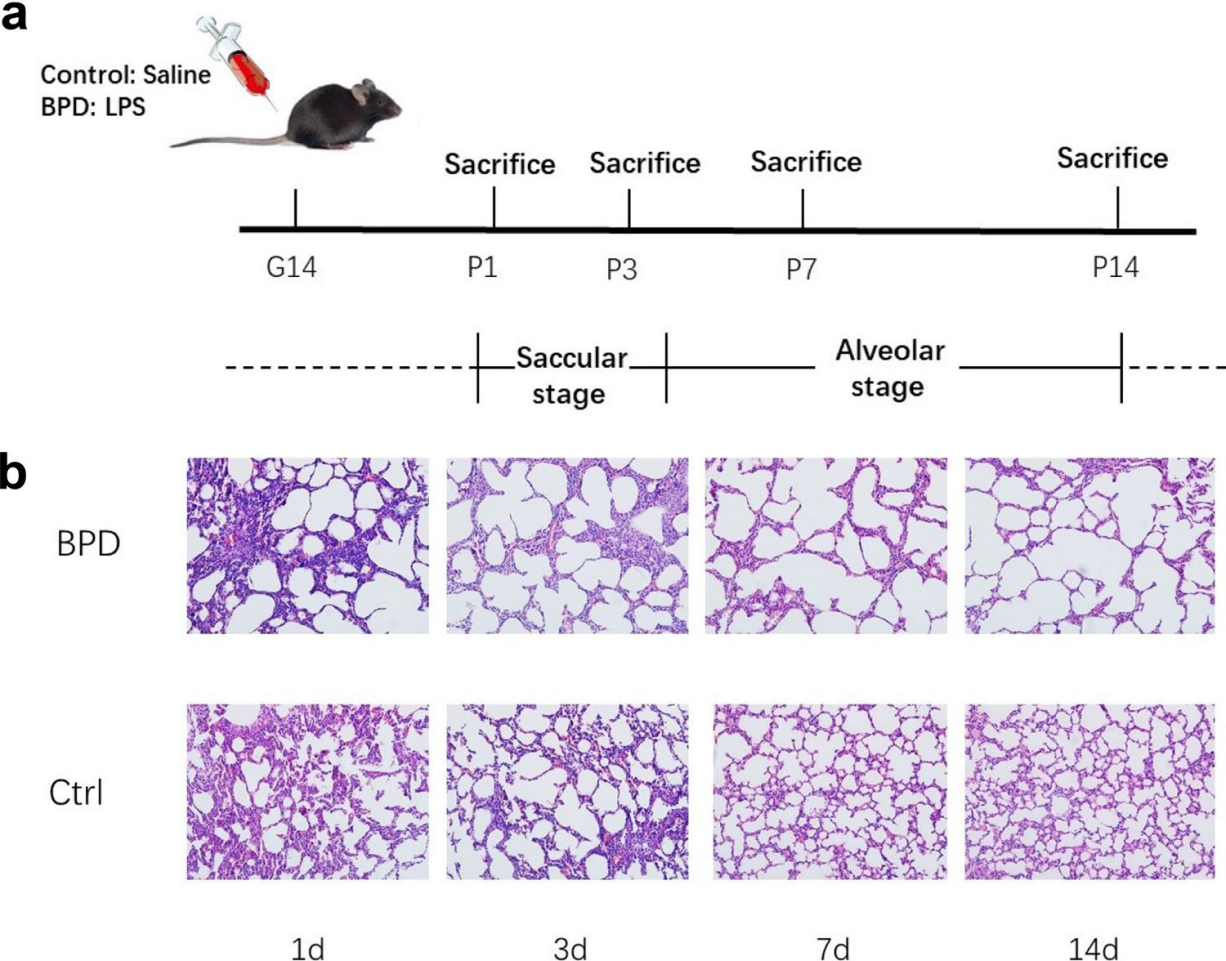
Experimental design for building animal model of BPD. **a** Pregnant mice (*n* = 5) were injected intraamnioticly with lipopolysaccharide on day 14 of pregnancy, and newborn mice were sacrificed on postnatal days 1, 3, 7, and 14. **b** Lung tissues were taken from mice of BPD and control group, stained with hematoxylin–eosin, and were observed by microscope. Original magnification × 200.

To investigate whether the BPD model was successful, we observed the morphology changes of lung tissues from BPD and control groups under a microscope. In the control group which had received normal saline, the alveolar structure became complete as well as the alveolar wall became thinner gradually. Simultaneously, in the BPD group which had received LPS, the alveolar number was decreased significantly, the alveolar structure was simplified, and inflammatory cells were apparent compared with the control group. Histology images of lung tissues from mice in the BPD and control groups are shown in Fig. 1.

### The Proportion of Nkp46^−^ILC3 in Lung Tissues Was Increased in BPD Mice

**Fig. 2:**
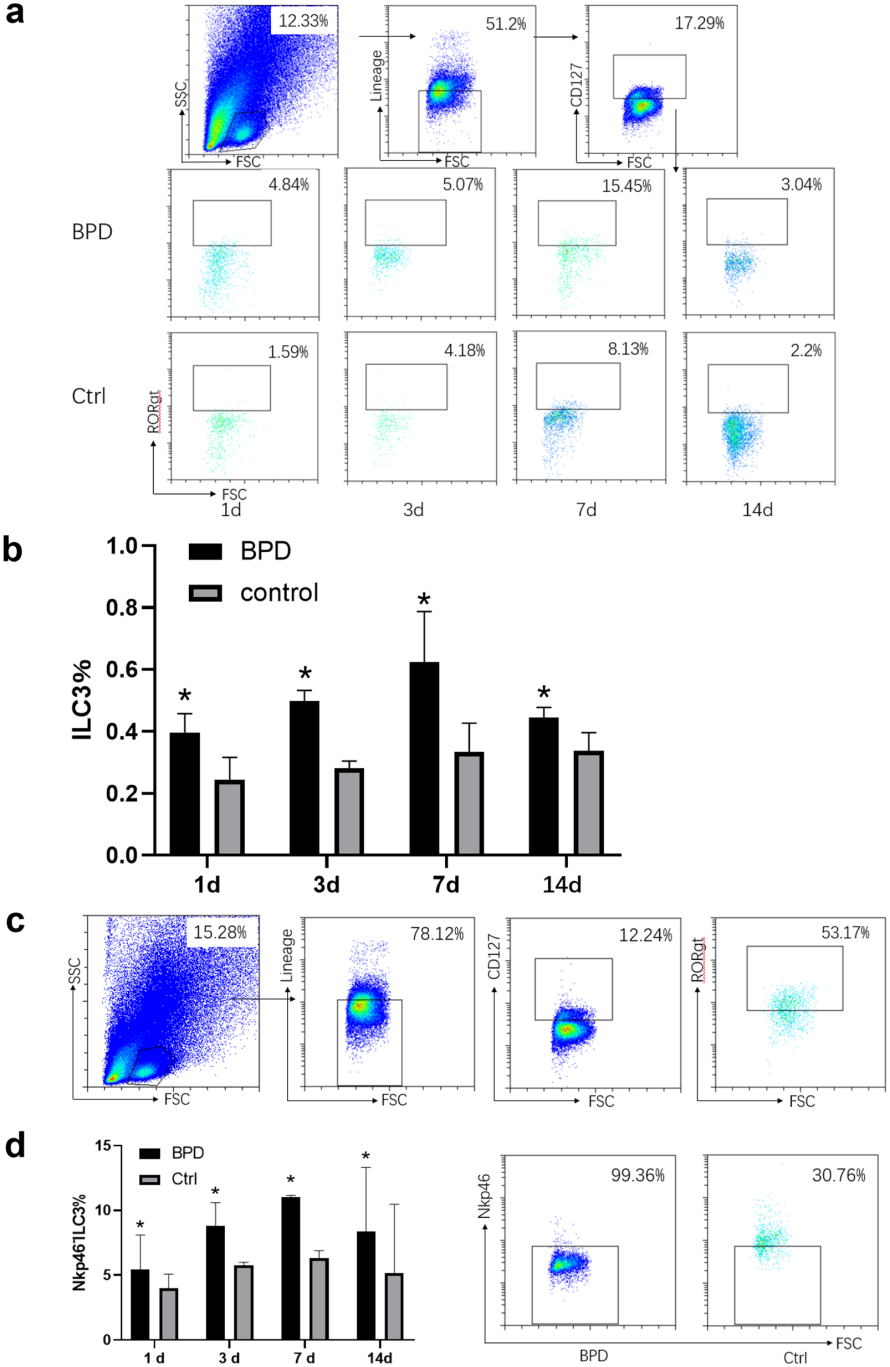
The proportion of ILC3 was increased lung tissues of BPD mice. **a** Mice of the BPD and control group were sacrificed on postnatal days 1, 3, 7, and 14. Percentage of ILC3 in lung tissues of mice in the BPD and control group was detected by flow cytometry. **b** Proportion of ILC3 in the BPD and control group (*n* = 5 for each group) at different time point. *P < 0.05 comparison among the BPD and control group. **c** Nkp46-ILC3 were presented as flow cytometry plots. **d** Quantification of Nkp46-ILC3 in the BPD and control group (*n* = 5 for each group) at different time point. *P < 0.05 comparison among the BPD and control group.

To explore the changes of ILC3, we detected the percentage of ILC3 in lung tissues from BPD mice by flow cytometry. In the control group, the proportion of ILC3 in lung tissues was increased gradually at 1, 3, 7, and 14 days after birth, reaching a peak on day 7, and then decreased gradually. The trend of ILC3 in BPD group was consistent with the control group. However, compared with the control group, the proportion of ILC3 in lung tissues of mice in BPD group was significantly increased (Fig. 2A, B). These results indicated that ILC3 may play a certain role in BPD. Based on the expression of Nkp46, ILC3 can be divided into two populations, namely NCR^**−**^ILC3 and NCR^+^ILC3. To examine which population predominantly works in BPD, single cell suspensions of lung tissues were stained with Nkp46 by flow cytometry. Compared with the control group, the expression of Nkp46^**+**^ILC3 in lung tissues of BPD group was significantly decreased (Fig. 2C, D). Taken together, these results provide evidence that the ILC3 subset at work in BPD may be the population of Nkp46^−^ILC3.

### The Increasing Expression of Cytokines Are Derived from ILC3

**Fig. 3:**
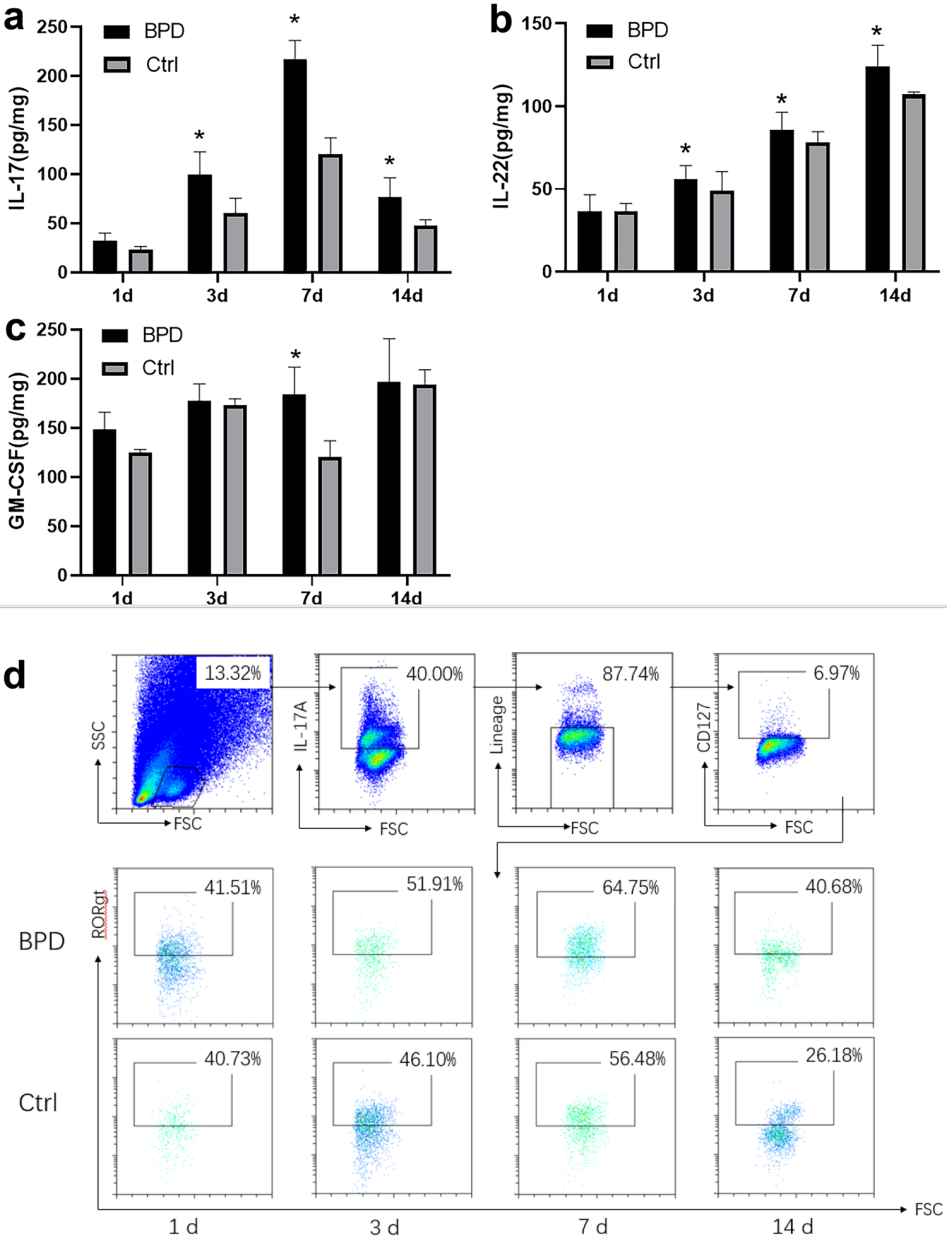
Expression of ILC3-derived IL-17 was increased in BPD mice.** a** Protein level of IL-17 in lungs of mice from the BPD and control group. **b** Protein level of IL-22 in lungs of mice from the BPD and control group. **c** Protein level of GM-CSF in lungs of mice from the BPD and control group. **d** Flow cytometry analysis of ILC3 cells among IL-17 + lymphocytes in the BPD and control group. Results are reported as group means ± SEM, with *n* = 5 for each group. *P < 0.05 comparison among the BPD and control group.

We collected lungs of mice on postnatal days 1, 3, 7, and 14 from control and BPD groups and measured protein level of IL-17, IL-22, and GM-CSF. After birth, the expression level of IL-17 in lung tissues of mice in control and BPD groups first rose and then fell. The expression of IL-17 showed an increasing trend at 1, 3, and 7 days after birth, reached the highest level on day 7, and showed apparent declination on day 14. Compared with the control group, the expression of IL-17 in the BPD group was increased significantly at the same time point, and the difference was statistically significant (*P* < 0.05) (Fig. 3A). The expression of IL-22 in control and BPD groups was increased gradually from day 1 to day 14. Compared with the control group, expression of IL-22 in the BPD group was increased at the same time point, with statistically significant difference (*P* < 0.05) (Fig. 3B). Compared with the control group, the trend of GM-CSF expression in the BPD group was not obvious from postnatal day 1 to day 14 (Fig. 3C). To study the origin of IL-17, we use flow cytometry to detect IL-17^+^ILC3. About 40% of lymphocytes in lung tissues of BPD mice expressed IL-17, and the proportion of ILC3 in the IL-17 ^+^ lymphocytes was 9%. Compared with the control group, the proportion of IL-17 ^+^ILC3 in the BPD group was increased at the same time point (*P* < 0.05) (Fig. 3D).

### ILC3 Aggravate Inflammation in BPD

**Fig. 4:**
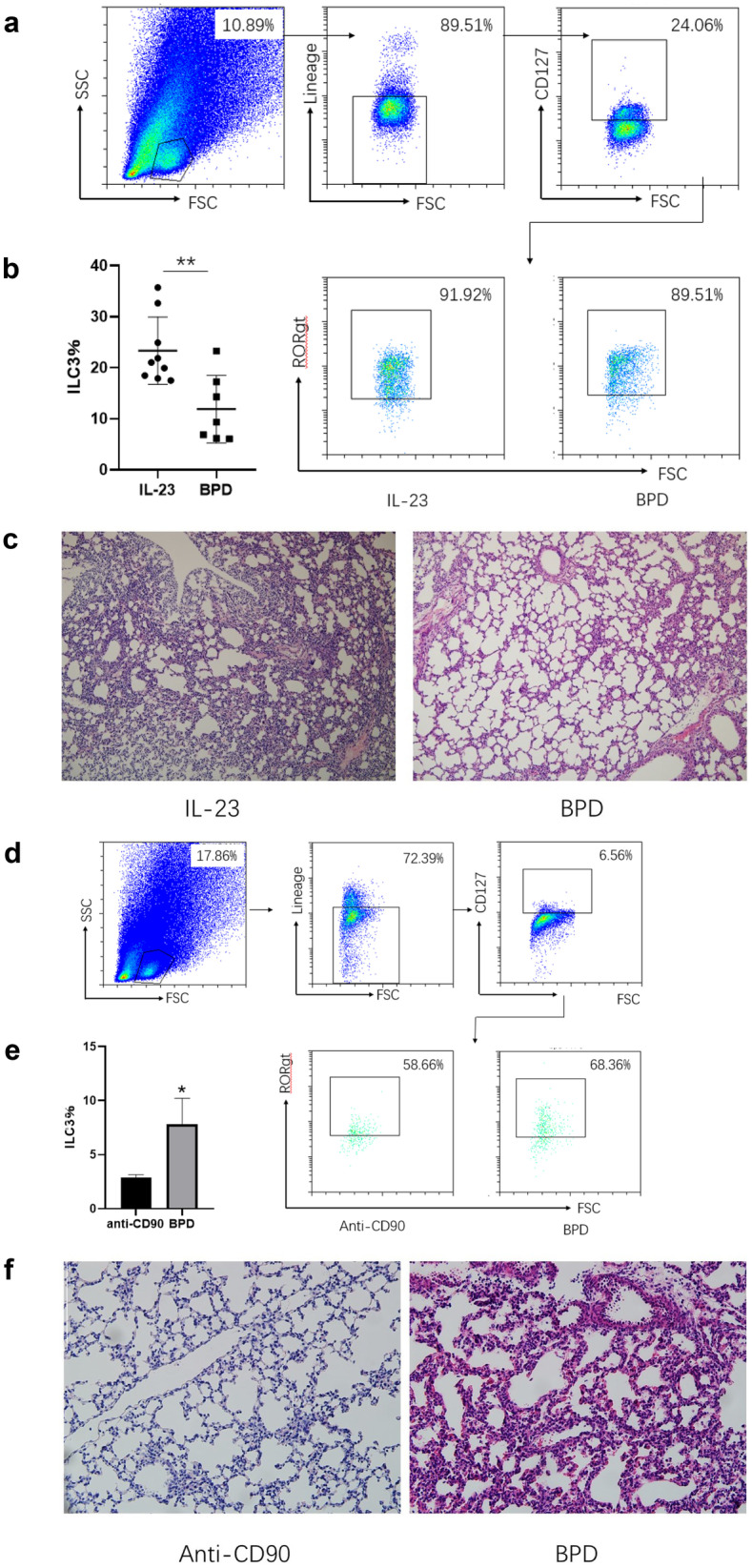
ILC3 aggravated inflammation in BPD.** a** Flow cytometry plots showing ILC3 in lung tissues of IL-23-treated mice and BPD mice. **b** Quantification of ILC3 in lung tissues of IL-23-treated mice and BPD mice (*n* = 5 for each group). **P < 0.01 comparison among the IL-23 treatment and BPD group. **c** Pathological observation of lung tissues from IL-23-treated mice and BPD mice. Original magnification × 200. **d** Flow cytometry analysis of ILC3 in anti-CD90 antibody-treated mice and BPD mice. **e** Quantification of ILC3 in lung tissues of anti-CD90 antibody-treated mice and BPD mice (*n* = 5 for each group). *P < 0.05 comparison among the anti-CD90 antibody treatment and BPD group. **f** Morphological changes of lung tissues from anti-CD90 antibody-treated mice and BPD mice. Original magnification × 200.

ILC3 from lungs of BPD mice express CD90 and RORγt, which are characteristics of ILC3. Studies have identified that IL-23 stimulates amplification of ILC3 [22]. To assess the specific role of ILC3 in BPD, we injected recombinant IL-23 into patient mice to stimulate ILC3 and then collected lung tissues to observe morphological changes after HE staining. Compared with the control group, lung tissues in the IL-23 treatment group showed more severe alveolar destruction and aggravated inflammation with the increase of ILC3 (Fig. 4A, B, C). These results suggested that ILC3 may play a pro-inflammatory role in BPD. To further investigate the role of ILC3 in BPD, mice were intraperitoneally injected with anti-CD90 antibody. We assessed whether ILC3 were knocked down using flow cytometry. The ILC3 ratio in lung tissues was detected, and the results showed that the ILC3 ratio in lung tissues of BPD mice treated with anti-CD90 antibody was significantly decreased compared with those treated with normal saline. (Fig. 4D, E). To evaluate the pathological changes of lung tissues after knockdown of ILC3, we selected the lung tissues of mice in the anti-CD90 antibody treatment group on day 14 for HE staining. Microscopic observation showed that compared with mice in the BPD group, mice in the anti-CD90 antibody treatment group had better pulmonary pathology, significantly reduced inflammation, and improved alveolar destruction (Fig. 4F). Therefore, the increase of ILC3 may aggravate the condition of BPD. These results suggested that ILC3 may play a destructive role in BPD.

### ILC3-Mediated Aggravation in BPD Is IL-17A Dependent

**Fig. 5:**
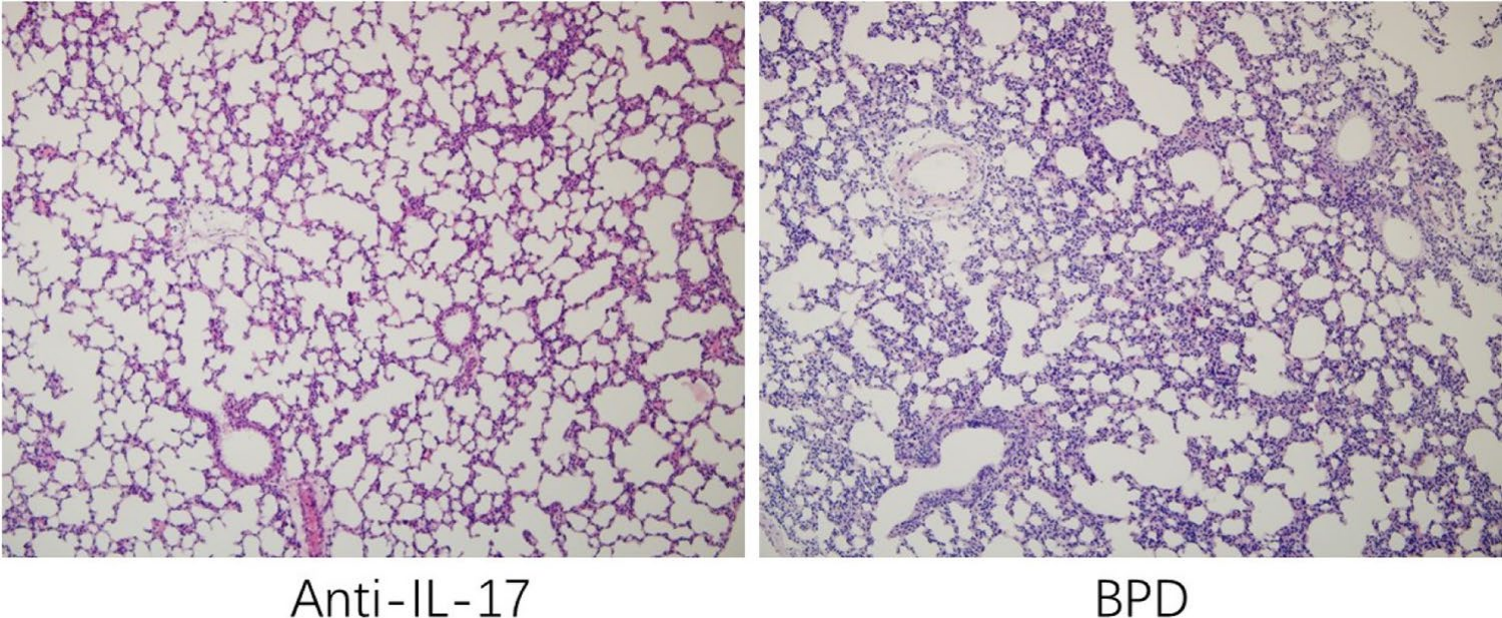
ILC3-mediated inflammation was dependent of IL-17. Pathological observation of lung tissues from anti-IL-17 antibody-treated mice and BPD mice. Original magnification × 200.

Studies have shown that ILC3 are a rapid source of IL-17 and play an important role in various inflammatory lung diseases. Thus, we hypothesized that the destructive effect mediated by ILC3 might depend on the production of IL-17. Mice were injected intraperitoneally with anti-IL-17A neutralizing antibody after birth, and lung tissues were taken for HE staining on postnatal day 14. Microscopic observation showed that compared with mice in the BPD group, mice in the anti-IL-17A neutralizing antibody treatment group had significantly reduced pulmonary inflammation and improved alveolar destruction (Fig. 5), indicating that mice lacking IL-17 had improved pulmonary pathology. Collectively, these results indicated that IL-17 derived from ILC3 may exacerbate pulmonary inflammation in BPD.

## DISCUSSION

ILC are a recently discovered lymphocyte population that mainly mediate mucosal immunity. Among them, ILC3 are involved in the regulation of various inflammatory lung diseases [14–17]. Inflammation is one of the most important pathogenic factors of BPD, so we aimed to investigate whether ILC3 and their downstream cytokines play a role in BPD.

In our study, LPS was injected into the amniotic cavity of C57BL/6 female mice on the 14th day of gestation to induce the occurrence of BPD in newborn mice. HE staining of lung tissues from newborn mice showed that the number of alveoli was decreased, the volume was increased, the alveolar structure was simplified, and the inflammation was aggravated; these pathological changes indicated that the BPD model was successful. In this model, we intended to explore the dynamic changes of ILC3. We found that the proportion of ILC3 in lung tissues of mice from the BPD group had changed. That is, the ILC3 ratio in lung tissues of newborn mice from the BPD group rose first on days 1, 3, and 7 and then decreased significantly on day 14. The trend of ILC3 ratio in the control group was consistent with the BPD group. Compared with the control group, the proportion of ILC3 in lung tissues of mice from the BPD group was increased at the same time point. Since ILC3 are involved in a variety of inflammatory lung diseases, and in combination with the changes of ILC3 in this model, we speculated that ILC3 might play a crucial role in BPD. This is consistent with the study that increase of ILC3 was found in sputum of noneosinophilic asthmatic patients in comparison with normal control [25]. In addition, since ILC3 can be divided into NCR^−^ILC3 and NCR^+^ILC3 according to the expression of Nkp46, in this study, we determined which population plays the predominant role in BPD by flow cytometry. Results showed that compared with the control group, the NKp46^−^ILC3 in the BPD group were significantly increased, namely NCR^−^ILC3. Previous studies have suggested that a fivefold increase of pro-inflammatory type 3 NKp46^−^ ILC3 was found in murine NEC model which may be correlated with the development of mouse necrotizing enterocolitis (NEC) [26]. Thus, it can be seen that NCR^−^ILC3 group plays a major role in BPD.

IL-23 is known to stimulate the proliferation of ILC3 [27]. To investigate the effect of ILC3 in BPD, the stimulation of ILC3 was performed by intraperitoneal injection of recombinant IL-23 into BPD mice. The proportion of ILC3 in lung tissues of mice from the IL-23 treatment group was detected by flow cytometry. Compared with the BPD group, the ILC3 ratio in lung tissues of mice from the IL-23 treatment group was significantly increased, and lung morphology was observed then. Several investigators found that the expression of ILC3 transcripts and frequency of IL-22 producing ILC3 was increased in response to IL-23 [28]. Compared with the BPD group, the number of alveoli of mice from the IL-23 treatment group was further decreased, the volume was further increased, and the inflammation was aggravated. These data suggested that with the increase of ILC3, the pulmonary inflammation of BPD is aggravated, indirectly indicating that ILC3 may play a crucial role in BPD. To further confirm the proinflammatory effect of ILC3, we knocked down ILC3 by intraperitoneal injection of anti-CD90 antibody into BPD mice [23]. Morphological observation of lung tissues in the anti-CD90 antibody treatment group showed that the severity of BPD was alleviated in the absence of ILC3. Previous studies have also demonstrated that ILC3 play an essential role in lung inflammation. For example, in obesity-induced asthma, ILC3 can release IL-17 in reaction to macrophage-derived IL-1β. ILC3 can also recruit neutrophils to lungs through IL-17, which leads to neutrophil asthma [22]. Taken together, these results demonstrated that ILC3 can aggravate BPD.

Studies have shown that ILC3 play a role in pulmonary diseases through the IL-17/IL-22 axis by rapidly secreting IL-17 and IL-22, which are involved in pulmonary inflammation and infectious diseases [5]. Moreover, IL-17 can act on a variety of cells, including epithelial cells, endothelial cells, and mesenchymal cells [29–31], so we wondered whether ILC3-derived IL-17 could affect BPD. In our study, we found that the expression of IL-17 in lung tissues of mice from BPD and control groups was increased gradually after birth, peaking on day 7, and then decreased. Compared with the control group, the expression of IL-17 in the BPD group was increased at the same time point, and the trend was consistent with changes of ILC3. These data suggested that IL-17 may correlate with the development of BPD. Hanashiro J et al. found that IL-17 expression is increased in salmonella-induced asthma model and plays a key role in asthma by mediating neutrophilic inflammation [32]. Besides, there were significant differences in the proportion of IL-17 ^+^ILC3 of lung tissues from the BPD and control group. Among lymphocytes expressing IL-17, the proportion of ILC3 was 9%. Compared with the control group, the proportion of IL-17 ^+^ILC3 was higher in the BPD group at the same time point. These results suggested that IL-17 secreted by ILC3 may play a role in BPD. Although small in number, ILC3 are a rapid source of IL-17 in inflammatory responses, and thus have the ability to drive inflammation. Furthermore, IL-17 can enhance the uptake and killing of bacteria by monocytes and plays an important role in a variety of bacterial pneumonia, including those caused by klebsiella and pseudomonas aeruginosa [33, 34]. IL-17, a key cytokine for accumulation and activation of neutrophils, can recruit neutrophils by inducing chemokines Cxcl1 and Cxxl9 [35]. It can regulate the migration of lung neutrophils, promote the generation of oxygen free radicals and extracellular traps in mouse neutrophils, and ultimately aggravate lung injury [36, 37]. Besides, IL-17 can also induce the production of various inflammatory cytokines, such as tumor necrosis factor (TNF), IL-6, and GM-CSF [38], suggesting that IL-17 plays an important role in inflammation. To further verify the role of IL-17 in BPD, intraperitoneal injection of anti-IL-17 neutralizing antibody was performed on BPD mice [24], and lung tissues were taken for HE staining to observe the pathological changes. It was found that the number of alveoli was increased, the volume was decreased, and the inflammation was alleviated, suggesting that the severity of BPD was relieved when lacking in IL-17. Taken together, these data demonstrated that ILC3 may recruit neutrophils by secreting IL-17, thereby aggravate the inflammation of BPD. In combination with the result that NKp46^−^ILC3 rather NKp46^+^ILC3 in the BPD group were significantly increased compared with the control group, we suggested that NKp46^−^ILC3 can aggravate the inflammation of BPD by secreting IL-17.

IL-22, which can be produced by ILC3, is involved in a variety of mucosa-related infectious and inflammatory lung diseases [39]. In our study, we found that the expression of IL-22 in lung tissues of mice from BPD and control groups was increased gradually from 1 to 7 days after birth. Compared with the control group, the expression of IL-22 in the BPD group was increased at the same time point, indicating that it may play a role in BPD. IL-22 appears to have an obvious function of tissue protection. By controlling the proliferation and differentiation of epithelial cells, it mediates the healing and regeneration of epithelial cells after injury, suggesting that it may play a restorative role in the later stage of injury. IL-22 can also improve the prognosis of mice by upregulating interferon-λ and inhibiting neutrophil recruitment to reduce lung epithelial injury in mouse model of pseudomonas aeruginosa pneumonia [40]. In summary, IL-22 may play a protective role in BPD.

This study showed that the expression of NCR^−^ILC3 and IL-17 exhibits changes in the development of BPD. Taken together, NCR^−^ILC3 may play a proinflammatory role by secreting IL-17. However, there may be some limitations in this experiment. The function of other subsets of ILC3 in pulmonary dynamic balance and lung diseases still needs further study. Moreover, whether other important immune cells in pulmonary immune environment could crosstalk with ILC3 can be the next research point.

## Data Availability

The data and materials used in this study are available from the corresponding author on reasonable request.

## References

[1] Revhaug C, Zasada M, Rognlien AGW, Gunther CC, Grabowska A, Ksiazek T (2020). Pulmonary vascular disease is evident in gene regulation of experimental bronchopulmonary dysplasia. The Journal of Maternal-Fetal & Neonatal Medicine.

[2] Morty RE (2018). Recent advances in the pathogenesis of BPD. Seminars in Perinatology.

[3] Ruegger C, Hegglin M, Adams M, Bucher HU, Swiss Neonatal N (2012). Population based trends in mortality morbidity and treatment for very preterm- and very low birth weight infants over 12 years. BMC Pediatrics.

[4] Islam JY, Keller RL, Aschner JL, Hartert TV, Moore PE (2015). Understanding the Short- and Long-Term Respiratory Outcomes of Prematurity and Bronchopulmonary Dysplasia. American Journal of Respiratory and Critical Care Medicine.

[5] Ardain A, Porterfield JZ, Kloverpris HN, Leslie A (2019). Type 3 ILCs in Lung Disease. Frontiers in Immunology.

[6] Bank U, Deiser K, Plaza-Sirvent C, Osbelt L, Witte A, Knop L (2020). c-FLIP is crucial for IL-7/IL-15-dependent NKp46(+) ILC development and protection from intestinal inflammation in mice. Nature Communications.

[7] Entwistle LJ, Puttur F, Gregory LG, Lloyd CM (2020). Group 2 ILC Functional Assays in Allergic Airway Inflammation. Methods in Molecular Biology.

[8] Geremia A, Arancibia-Carcamo CV (2017). Innate Lymphoid Cells in Intestinal Inflammation. Frontiers in Immunology.

[9] Reeves RK, De Grove KC, Provoost S, Verhamme FM, Bracke KR, Joos GF (2016). Characterization and Quantification of Innate Lymphoid Cell Subsets in Human Lung. PLoS ONE.

[10] Diefenbach A, Colonna M, Koyasu S (2014). Development differentiation and diversity of innate lymphoid cells. Immunity.

[11] Zeng B, Shi S, Ashworth G, Dong C, Liu J, Xing F (2019). ILC3 function as a double-edged sword in inflammatory bowel diseases. Cell Death & Disease.

[12] van de Pavert SA, Vivier E (2016). Differentiation and function of group 3 innate lymphoid cells from embryo to adult. International Immunology.

[13] Rankin LC, Girard-Madoux MJ, Seillet C, Mielke LA, Kerdiles Y, Fenis A (2016). Complementarity and redundancy of IL-22-producing innate lymphoid cells. Nature Immunology.

[14] Eken A, Singh AK, Treuting PM, Oukka M (2014). IL-23R+ innate lymphoid cells induce colitis via interleukin-22-dependent mechanism. Mucosal Immunology.

[15] Song C, Lee JS, Gilfillan S, Robinette ML, Newberry RD, Stappenbeck TS (2015). Unique and redundant functions of NKp46+ ILC3s in models of intestinal inflammation. Journal of Experimental Medicine.

[16] Steigler P, Daniels NJ, McCulloch TR, Ryder BM, Sandford SK, Kirman JR (2018). BCG vaccination drives accumulation and effector function of innate lymphoid cells in murine lungs. Immunology and Cell Biology.

[17] Maertzdorf J, Tonnies M, Lozza L, Schommer-Leitner S, Mollenkopf H, Bauer TT (2018). Mycobacterium tuberculosis Invasion of the Human Lung: First Contact. Frontiers in Immunology.

[18] Everaere L, Ait-Yahia S, Molendi-Coste O, Vorng H, Quemener S, LeVu P (2016). Innate lymphoid cells contribute to allergic airway disease exacerbation by obesity. Journal of Allergy and Clinical Immunology.

[19] Ambigapathy G, Schmit T, Mathur RK, Nookala S, Bahri S, Pirofski LA (2019). Double-Edged Role of Interleukin 17A in Streptococcus pneumoniae Pathogenesis During Influenza Virus Coinfection. The Journal of Infectious Diseases.

[20] Wu Y, Yue J, Wu J, Zhou W, Li D, Ding K (2018). Obesity May Provide Pro-ILC3 Development Inflammatory Environment in Asthmatic Children. Journal of Immunology Research.

[21] Yanagisawa H, Hashimoto M, Minagawa S, Takasaka N, Ma R, Moermans C (2017). Role of IL-17A in murine models of COPD airway disease. American Journal of Physiology-Lung Cellular and Molecular Physiology.

[22] Jonckheere AC, Bullens DMA, Seys SF (2019). Innate lymphoid cells in asthma: Pathophysiological insights from murine models to human asthma phenotypes. Current Opinion in Allergy and Clinical Immunology.

[23] Xiong H, Keith James W, Samilo Dane W, Carter Rebecca A, Leiner Ingrid M, Pamer Eric G (2016). Innate Lymphocyte/Ly6C hi Monocyte Crosstalk Promotes Klebsiella Pneumoniae Clearance. Cell.

[24] Liu M, Zhao Y, Wang C, Luo H, Peng A, Ye L (2019). Interleukin-17 plays a role in pulp inflammation partly by WNT5A protein induction. Archives of Oral Biology.

[25] Kim J, Chang Y, Bae B, Sohn KH, Cho SH, Chung DH (2019). Innate immune crosstalk in asthmatic airways: Innate lymphoid cells coordinate polarization of lung macrophages. Journal of Allergy and Clinical Immunology.

[26] Cho SX, Rudloff I, Lao JC, Pang MA, Goldberg R, Bui CB (2020). Characterization of the pathoimmunology of necrotizing enterocolitis reveals novel therapeutic opportunities. Nature Communications.

[27] Liu Y, Song Y, Lin D, Lei L, Mei Y, Jin Z (2019). NCR(-) group 3 innate lymphoid cells orchestrate IL-23/IL-17 axis to promote hepatocellular carcinoma development. eBioMedicine.

[28] Mazzurana L, Forkel M, Rao A, Van Acker A, Kokkinou E, Ichiya T (2019). Suppression of Aiolos and Ikaros expression by lenalidomide reduces human ILC3-ILC1/NK cell transdifferentiation. European Journal of Immunology.

[29] Mai J, Nanayakkara G, Lopez-Pastrana J, Li X, Li YF, Wang X (2016). Interleukin-17A Promotes Aortic Endothelial Cell Activation via Transcriptionally and Post-translationally Activating p38 Mitogen-activated Protein Kinase (MAPK) Pathway. Journal of Biological Chemistry.

[30] Wang Z, Jia Y, Du F, Chen M, Dong X, Chen Y (2017). IL-17A Inhibits Osteogenic Differentiation of Bone Mesenchymal Stem Cells via Wnt Signaling Pathway. Medical Science Monitor.

[31] Luo J, An X, Yao Y, Erb C, Ferguson A, Kolls JK (2019). Epigenetic Regulation of IL-17-Induced Chemokines in Lung Epithelial Cells. Mediators of Inflammation.

[32] Hanashiro J, Muraosa Y, Toyotome T, Hirose K, Watanabe A, Kamei K (2019). Schizophyllum commune induces IL-17-mediated neutrophilic airway inflammation in OVA-induced asthma model mice. Scientific Reports.

[33] Bayes HK, Ritchie ND, Evans TJ (2016). Interleukin-17 Is Required for Control of Chronic Lung Infection Caused by Pseudomonas aeruginosa. Infection and Immunity.

[34] Chuammitri P, Wongsawan K, Pringproa K, Thanawongnuwech R (2019). Interleukin 17 (IL-17) manipulates mouse bone marrow- derived neutrophils in response to acute lung inflammation. Comparative Immunology Microbiology and Infectious Diseases.

[35] Kuwabara T, Ishikawa F, Kondo M, Kakiuchi T (2017). The Role of IL-17 and Related Cytokines in Inflammatory Autoimmune Diseases. Mediators of Inflammation.

[36] Ma WT, Gu K, Yang R, Tang XD, Qi YX, Liu MJ (2020). Interleukin-17 mediates lung injury by promoting neutrophil accumulation during the development of contagious caprine pleuropneumonia. Veterinary Microbiology.

[37] Li Y, Shen Y, Lin D, Zhang H, Wang T, Liu H (2019). Neutrophils and IL17A mediate flagellar hook protein FlgE-induced mouse acute lung inflammation. Cellular Microbiology.

[38] Zong S, Li K, Zeng G, Fang Y, Zhao J (2016). The Effects of Interleukin-17 (IL-17)-Related Inflammatory Cytokines and A20 Regulatory Proteins on Astrocytes in Spinal Cord Cultured In Vitro. Cellular Physiology and Biochemistry.

[39] Hebert KD, McLaughlin N, Galeas-Pena M, Zhang Z, Eddens T, Govero A (2020). Targeting the IL-22/IL-22BP axis enhances tight junctions and reduces inflammation during influenza infection. Mucosal Immunology.

[40] Broquet A, Besbes A, Martin J, Jacqueline C, Vourc'h M, Roquilly A (2020). Interleukin-22 regulates interferon lambda expression in a mice model of pseudomonas aeruginosa pneumonia. Molecular Immunology.

